# Sexual behaviour

**DOI:** 10.1016/j.mpmed.2014.03.005

**Published:** 2014-06

**Authors:** Catherine H. Mercer

**Affiliations:** **Catherine H Mercer BSc (Soc Sci) MSc (Soc Sci) PhD** is a Senior Lecturer in the Centre for Sexual Health and HIV Research at University College London, London, UK. Conflicts of interest: none declared

**Keywords:** Heterosexual, homosexual, sexual behaviour, sexual health, sexually transmitted infection

## Abstract

Sexual health is not merely the absence of disease, but the ability to have informed, consensual, safe, respectful, and pleasurable sexual relationships. The majority of the population are sexually active, most with someone of the opposite sex. The frequency and range of sexual practices that people engage in declines with age, but for many, sexual activity continues well into later life. Different aspects of sexual health affect people at different times throughout their lives. As people in the UK tend to first have sex around the age of 16, but do not start living with a partner until much later, the avoidance of sexually transmitted infections and unplanned pregnancy is necessary for many for a number of years. As people get older, their sexual health needs change and they become more concerned with the impact of their general health on their ability to have sex. Some people experience non-volitional sex (sex against their will); although this occurs typically in late teenage it may affect women *and* men at any age and so requires consideration throughout life. As many people find it difficult to talk about sex and sexual health matters, health professionals should make sexual health enquiry a component of their holistic healthcare.

**What's new?**

• Sexual health is increasingly recognized as not merely the absence of disease, but the ability to have pleasurable and safe sexual experiences, free from coercion
• Sexual health is important throughout life, although sexual health needs vary with age
• Many people find it difficult to talk about sex; health professionals need to be aware of this and prepared to raise the subject

In recent years, there has been a shift in how sexual health is conceptualized.^1^, ^2^ No longer is it concerned solely with sexually transmitted infections (STIs) or avoiding unplanned pregnancy; it is increasingly recognized as additionally encompassing elements of broader reproductive health, sexual function, and non-volitional sex, and is therefore important throughout the lifecourse. Understanding sexual health requires an understanding of sexual behaviour, yet this is seldom discussed in the consultation room, despite sexual relationships being fundamental to individual, family and social life in all cultures, and there being interplay between an individual's general health and their sexual health.^3^

The scientific study of sexual behaviour has come a long way since the ground-breaking, but methodologically problematic surveys undertaken by Kinsey in the 1940s and 1950s.^4^, ^5^ In Britain, a large-scale, probability sample survey of sexual behaviour – the National Survey of Sexual Attitudes and Lifestyles (Natsal) – has been undertaken every ten years since 1990.^6^, ^7^, ^8^, ^9^, ^10^, ^11^ As Natsal surveys are broadly representative of the British general population, they are regarded as important resources for health service planning, epidemiology and understanding of sexual behaviour. This article draws heavily on the most recent Natsal survey, and so gives up-to-date insights into the sexual lifestyles of the British population.

## Sexual behaviour with partners of the opposite sex

**Figure 1:**
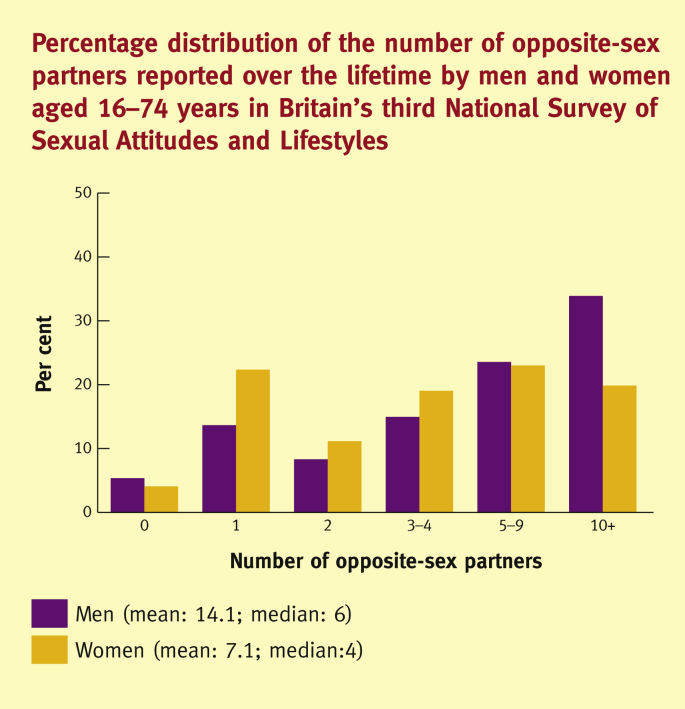
Percentage distribution of the number of opposite-sex partners reported over the life time by men and women aged 16–74 years in Britain's third National Survey of Sexual Attitudes and lifestyles (‘Natsal-3’).

**Figure 2:**
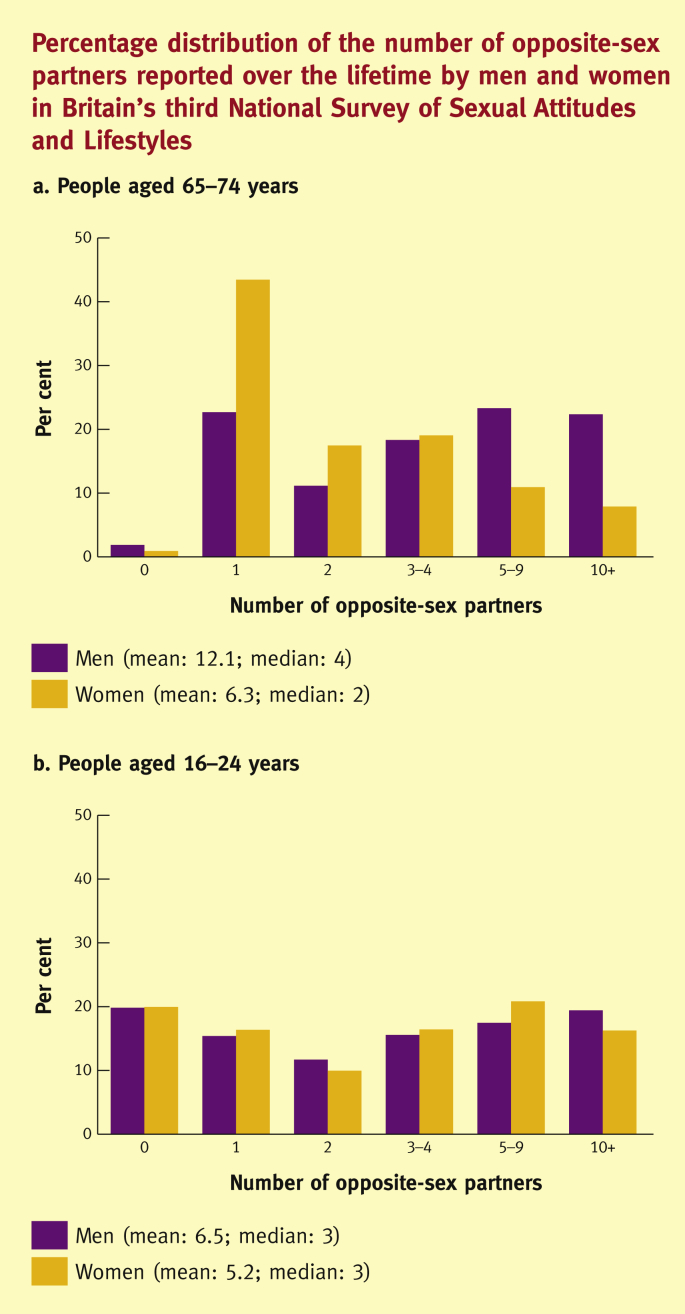
Percentage distribution of the number of opposite-sex partners reported over the life time by men and women in Britain's third National Survey of Sexual Attitudes and lifestyles (‘Natsal-3’).

In Britain, 82% of men and 78% of women aged 16–74 years have had sex in the past year with at least one partner of the opposite sex, although this declines significantly with age.^12^ Around 20% of men and 15% of women have had new opposite-sex partner(s) in the past year, although these percentages are significantly higher among young people (those aged 16–24 years) at 46% and 38%, respectively.^12^ Whereas many people in the population have few sexual partners – regardless of the time-frame considered – few people have many sexual partners. Among all people aged 16–74, one in three men and one in four women have had at least ten opposite-sex partners over their life time (Figure 1).^12^ Previously,^6^, ^7^, ^8^, ^9^ there was a large gap between the numbers of sexual partners reported by men and women, but in recent years this gender gap has narrowed considerably and there is now no difference in the numbers of sexual partners reported by young men and women (Figure 2).^12^

Vaginal sex is the mainstay of the heterosexual repertoire, experienced at least once in the past month by around 60% of all men and women aged 16–74 years.^12^ Orogenital contact, as well as other genital contact not leading to intercourse (e.g. mutual masturbation), are experienced at least once in the past year by similar proportions of the population.^12^ While there have been increases in the reporting of experiencing heterosexual anal sex, it remains a relatively infrequent heterosexual practice (approximately one in ten men and women reported it at least once in the past year).^12^ However this rise in prevalence of anal sex may be very relevant for STI/HIV transmission, as transmission probabilities are high for this particular sexual practice.

Among those who have had sex in the past year, the median frequency of sexual intercourse in 16–74-year-old men and women is three times per month, with an inter-quartile range of 1 to 6 times, again reflecting the variability of sexual behaviour in the population.^12^ While sexual frequency declines with increasing age to a median of once a month among 65–74-year-olds, this figure serves as a reminder that sexual activity continues into older age for many people.

Poor health can impact on a person's sex life at any age. In Britain, 17% of men and women aged 16–74 years perceive that they have a health condition that affects their sex life.^3^ However, less than one-quarter of these people have sought help from a health professional. This signals a need to raise awareness, improve guidance, and build communication skills among health professionals so they are able to discuss sex with their patients as and when appropriate.

## Sexual behaviour with partners of the same sex

Approximately 3% of men and women in Britain have had same-sex partner(s) in the past 5 years.^12^ Men who have sex with men (MSM) are at particularly high risk of acquiring HIV and STIs. Anal intercourse – especially when a condom is not used (‘unprotected anal intercourse’) – is the most significant risk activity. Approximately two-thirds of MSM report anal sex in the past year, and of these, 40% always and 25% sometimes used a condom for this practice.^13^ Certain behavioural interventions, including individual counselling, small group and community interventions, have been shown to reduce rates of unprotected anal intercourse in this group.^14^

Women who have sex with women (WSW) have been less extensively studied, possibly because they tend to be at lower risk of STIs from having sex with women. However, WSW are still at risk of STIs as a large proportion of WSW also have sex with men, and those that do report larger numbers of male sexual partners than women who only have sex with men.^15^ It is important therefore to consider not merely a person's sexual identity, but also the sexual relations they have, and with whom.

## Sexual health needs during the whole of life

Nowadays, on average, men and women in Britain start having sex around age 16, whereas for previous generations, sexual debut occurred two or three years later than this.^12^ Whereas the age of consent is 16 years in the UK, English law states that contraceptives may be prescribed to those under 16 without parental consent under certain conditions (‘Gillick competence’), and the General Medical Council advises that minors are entitled to confidential consultation. Nevertheless, locally agreed child protection procedures must be followed when there are concerns about child sexual abuse (e.g. when one partner is much older than the other).

As well as an earlier age at sexual debut, nowadays people are not living with a partner until much later, and are also not having children until later.^16^ These demographic changes mean that many people now spend a greater amount of time needing to avoid unplanned pregnancy as well as STIs, which are most prevalent, but do not occur exclusively, among young people.^17^ However, even when couples are in a stable relationship, the need to avoid unplanned pregnancy continues well into their 40s because they are having fewer children.^16^ As people enter older age, health problems – and sexual response problems – become more prevalent,^3^, ^18^ so that sexual health becomes more about sexual function and the ability to maintain a satisfying sex life.

Non-volitional sex, or sex against a person's will, is a sexual health issue regardless of age, and is reported by 10% of all women and 1% of all men.^19^ Of those who reported having had sex against their will, only 42% of women and 33% of men had told anyone about it. Experiencing non-volitional sex was found to be associated with a range of poor physical, mental and sexual health problems, including depression requiring treatment. These data emphasise the need to remove the barriers that prevent people from talking about their experience and seeking help. This includes training health and other professionals to enable them to provide appropriate services and guidance for people, and to work together across different sectors to help mitigate the harm caused by these experiences.

Positive sexual experiences are related to health and wellbeing throughout life, so there is a need to think about sex differently in clinical practice, education, and research. Sexual health is not merely the absence of disease, but the ability to have pleasurable and safe sexual experiences, free from coercion. Improving the quality of peoples' sexual experiences and their relationships will not merely improve the effectiveness of sexual health programmes, but is also important in its own right.

**Practice points**

• Health professionals need to enquire about sexual health, desires and experience, including non-volitional sex, as many people are reluctant to talk about these matters
• Same-sex experience is reported by a significant minority of both men and women. Never assume the gender of a patient's partner or a past relationship
• Sexual repertoire can be very varied. Practitioners need to ask about this to be able to offer a complete risk assessment, appropriate testing and tailored risk-reduction advice
